# Frequency‐dependent selection: a diversifying force in microbial populations

**DOI:** 10.15252/msb.20167133

**Published:** 2016-08-03

**Authors:** Daniel A Charlebois, Gábor Balázsi

**Affiliations:** ^1^ The Louis and Beatrice Laufer Center for Physical & Quantitative Biology and Department of Biomedical EngineeringStony Brook UniversityStony BrookNYUSA

**Keywords:** Evolution, Microbiology, Virology & Host Pathogen Interaction, Quantitative Biology & Dynamical Systems

## Abstract

The benefits of “bet‐hedging” strategies have been assumed to be the main cause of phenotypic diversity in biological populations. However, in their recent work, Healey *et al* (2016) provide experimental support for negative frequency‐dependent selection (NFDS) as an alternative driving force of diversity. NFDS favors rare phenotypes over common ones, resulting in an evolutionarily stable mixture of phenotypes that is not necessarily optimal for population growth.

“E Pluribus Unum”—stands on the Great Seal of the United States, summarizing the benefits of diversity when “out of many, one” community forms. The same wisdom echoes in the current motto of the European Union, “In Varietate Concordia” meaning “United in diversity”. Notably, cell populations have been exploiting the benefits of unified diversity long before humans appeared on Earth. Cellular diversity in a population provides resilience and adaptability to changing environmental conditions. It has contributed to maintaining microbial life over geological time scales and it plays a central role in promoting the evolution of cancer and drug‐resistant infections (Charlebois *et al*, 2011; Pisco *et al*, 2013), which constitute major issues in patient treatments. While the evolutionary importance of heritable diversity has been known since Darwin, its causes and mechanisms, especially at the cellular level, are far from understood. The days when heritable cellular diversity could simply be attributed to genetic differences among cells are long gone. Environmental and stochastic effects are now considered to be equally important sources of cellular diversity (Kærn *et al*, 2005).

Is non‐genetic diversity merely a by‐product of microscopic life, in which environmental and internal molecular differences cause different cellular behaviors? The regulatory mechanisms that cells employ and evolve to modulate their diversity (Quan *et al*, 2012; González *et al*, 2015) seem to suggest otherwise. A common assumption has been that cells minimize diversity to take full advantage of stable, nutrient‐rich environments, but amplify diversity when the environment unexpectedly fluctuates and can potentially become harmful. For example, rare growth‐arrested persister cells can survive antibiotic treatment and eventually rescue bacterial populations, just like diversifying one's financial portfolio by investing in multiple unpredictably fluctuating stocks can protect from bankruptcy. While such “bet‐hedging” strategies have so far been assumed to explain most cellular diversity, a recent study (Healey *et al*, 2016) provides experimental support for negative frequency‐dependent selection (NFDS) as an alternative driving force of cellular diversity with no apparent benefits at the population level.

NFDS occurs when the fitness of a phenotype in the population decreases with its prevalence. For example, when two cell types specialize to use different resources from the same environment, their fitness is frequency dependent: More resource is available for the rare phenotype, which can produce more offspring per capita. As cells with the rare phenotype can spread (invade) among more frequent ones (Fig 1A), neither cell type can dominate. This generates an evolutionarily stable mix of phenotypes (diversity) without any benefits for the population. While NFDS has been suggested to cause non‐genetic cellular diversity (Rainey *et al*, 2000), Healey *et al* are the first to test this idea experimentally.

**Figure 1:**
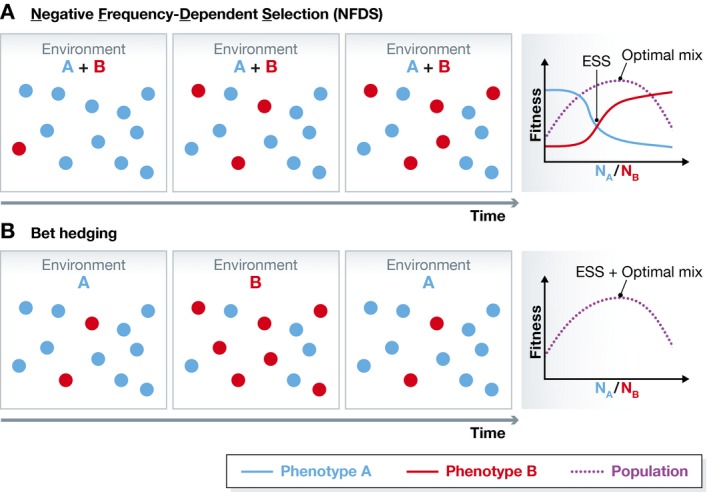
Cellular diversity arising from bet hedging versus negative frequency‐dependent selection (A) In negative frequency‐dependent selection, phenotypes are mutually invasible due to the advantage of rare phenotypes, for example, in environments with multiple resources. In this case, the evolutionarily stable mix of phenotypes does not maximize population fitness (growth rate). Maximal population fitness would require more cells of phenotype A growing on higher‐quality resource A. However, when phenotype A is prevalent, then phenotype B has a growth advantage because it has access to more resource B. Overall, phenotypic diversity emerges as phenotypes equalize their fitnesses and the population approaches the evolutionarily stable strategy (ESS), irrespective of the population‐level fitness. (B) In bet hedging, cells diversify spontaneously without directly sensing the environmental fluctuations that alternately select for distinct phenotypes. Bet‐hedging diversity is suboptimal in each constant environment, but optimizes population fitness in changing environments according to the rates of environmental fluctuations.

Environments containing both glucose and galactose induce activation of the native yeast GAL network in some but not all cells (Biggar & Crabtree, 2001). Thus, clonal yeast cells naturally separate into specialists that consume only glucose and those that consume both sugars. Healey *et al* (2016) modified the native GAL network to obtain obligate GAL‐OFF cells that only consume glucose and obligate GAL‐ON cells that consume both sugars. By co‐culturing obligate GAL‐ON and GAL‐OFF cells at various initial frequencies, the authors found that the two cell types mutually invaded each other and reached a stable mix in environments containing both glucose and galactose. GAL‐ON and GAL‐OFF cells had equal fitness in this stable mix while the population fitness was non‐optimal (a hallmark of NFDS, Fig 1A). Furthermore, cells with a wild‐type GAL network could invade both obligate cell types, but were uninvasible by either, indicating that the native GAL network enables a mix of galactose utilization phenotypes that is evolutionarily stable but non‐optimal. This is somewhat puzzling because natural selection should in principle tend to optimize the clonal population's fitness in a constant environment. On the other hand, deviations from optimality may be expected when fluctuating environments create changing fitness landscapes with shifting optima, or when species get stuck in local optima. Finally, Healey *et al* performed long‐term evolution of pure obligate GAL‐OFF or GAL‐ON cell populations and observed, in both cases, the emergence of a mixed GAL‐ON/GAL‐OFF population in mixed sugar conditions (but not in single‐sugar conditions). These novel examples of evolving phenotypic diversity support NFDS as an evolutionary driving force of cellular diversification in a relatively stable environment. In contrast to NFDS, bet‐hedging diversity requires alternating selection pressures (Beaumont *et al*, 2009) or else it declines in constant environments (González *et al*, 2015).

Healey *et al* discover one of potentially many evolutionary causes of cellular diversification. So can we distinguish NFDS from bet hedging and other possible sources of phenotypic diversity? Some differences might seem immediately apparent. Bet‐hedging diversity is generated spontaneously and randomly, irrespective of the environment, unlike the NFDS diversity discussed here, which manifested only in dual‐sugar environments. Bet hedging is beneficial in fluctuating environments (Acar *et al*, 2008), optimizing the overall population growth rate (Fig 1B), whereas NFDS merely equalizes the fitness of different phenotypes irrespective of population fitness. However, to avoid comparing apples and oranges, we should also realize how the fluctuating environments that promote bet hedging differ fundamentally from the stable, multi‐resource NFDS conditions. Thus, a deeper challenge may be to elucidate which of the distinguishing features of NFDS stem from the presence or lack of environmental fluctuations.

One way to reconcile these differences is to imagine tuning the rate of environmental fluctuations from ultra‐rapid to utterly slow. Then cells would probably perceive extremely fast glucose–galactose fluctuations as a mixture of both sugars and would diversify to obey NFDS. Extremely slow fluctuations would mimic constant environments and should diminish diversity (González *et al*, 2015). Somewhere in the middle between these extremes, bet hedging may occur. While Healey *et al* evolved diversity at one extreme (mixed sugars), one could test whether cells engineered for obligate diversity would become uniform in constant, single‐sugar environments. Moreover, it would be interesting to test whether obligate GAL‐ON or GAL‐OFF cells diversify in conditions that switch repeatedly, but not very frequently between glucose‐only and galactose‐only (Quan *et al*, 2012). In principle, the same phenotypic diversity could satisfy both NFDS and bet‐hedging criteria in different environmental fluctuation regimes. Overall, the driving forces of phenotypic cellular diversification should be environment dependent.

In summary, the discovery that NFDS can promote phenotypic cellular heterogeneity greatly broadens our view, but also generates many new questions. It will be interesting to see to what extent NFDS drives the myriad forms of phenotypic heterogeneity that are increasingly considered a confounding factor in treating drug‐resistant infections and cancers (Charlebois *et al*, 2011; Pisco *et al*, 2013).
